# Incidence Rates of Enterovirus 71 Infections in Young Children during a Nationwide Epidemic in Taiwan, 2008–09

**DOI:** 10.1371/journal.pntd.0001476

**Published:** 2012-02-14

**Authors:** Min-Shi Lee, Pai-Shan Chiang, Shu-Ting Luo, Mei-Liang Huang, Guan-Yuan Liou, Kuo-Chien Tsao, Tzou-Yien Lin

**Affiliations:** 1 National Institute of Infectious Diseases and Vaccinology, National Health Research Institutes, Zhunan, Taiwan; 2 Department of Medical Biotechnology and Laboratory Science, Chang Gung University, Taoyuan, Taiwan; 3 Department of Laboratory Medicine, Chang Gung Memorial Hospital, Taoyuan, Taiwan; 4 Department of Pediatrics, Chang Gung Children's Hospital, Taoyuan, Taiwan; University of Washington, United States of America

## Abstract

**Objective:**

Enterovirus 71 (EV71) is causing life-threatening outbreaks in tropical Asia. In Taiwan and other tropical Asian countries, although nationwide EV71 epidemics occur cyclically, age-specific incidence rates of EV71 infections that are critical to estimate disease burden and design vaccine trials are not clear. A nationwide EV71 epidemic occurred in 2008–09 in Taiwan, which provided a unique opportunity to estimate age-specific incidence rates of EV71 infections.

**Study Design:**

We prospectively recruited 749 healthy neonates and conducted follow-ups from June 2006 to December 2009. Sera were obtained from participants at 0, 6, 12, 24, and 36 months of age for measuring EV71 neutralizing antibody titers. If the participants developed suspected enterovirus illnesses, throat swabs were collected for virus isolation.

**Results:**

We detected 28 EV71 infections including 20 symptomatic and 8 asymptomatic infections. Age-specific incidence rates of EV71 infection increased from 1.71 per 100 person-years at 0–6 months of age to 4.09, 5.74, and 4.97 per 100 person-years at 7–12, 13–24, and 25–36 months of age, respectively. Cumulative incidence rate was 15.15 per 100 persons by 36 months of age, respectively.

**Conclusions:**

Risk of EV71 infections in Taiwan increased after 6 months of age during EV71 epidemics. The cumulative incidence rate was 15% by 36 months of age, and 29% of EV71 infections were asymptomatic in young children.

## Author Summary

Enterovirus 71 (EV71) was first isolated in California, USA, in 1969. Since then, EV71 has been identified globally. Recently, EV71 caused several life-threatening outbreaks in young children in tropical Asia. Development of EV71 vaccines becomes national priority in several Asia countries including Taiwan. To design clinical trials of EV71 vaccines, age-specific incidence rates of EV71 infections are required to identify target populations, estimate disease burdens, select endpoints of clinical efficacy, and estimate sample size. In Taiwan, nationwide EV71 epidemics occurred every 3–4 years but age-specific incidences of EV71 infection are not available. In 2006, we initiated a prospective cohort study in northern Taiwan to recruit neonates and follow up them. In 2008–09, a nationwide EV71 epidemic occurred and we found that age-specific incidence rates of EV71 infection increased from 1.71 per 100 person-years at 0–6 months of age to 4.09, 5.74, and 4.97 per 100 person-years at 7–12, 13–24, and 25–36 months of age, respectively. The cumulative incidence rate was 15% by 36 months of age, and 29% of EV71 infections were asymptomatic in young children. These findings would be helpful to development of EV71 vaccines in Taiwan and other Asian tropical countries.

## Introduction

Enterovirus 71 (EV71) was first isolated in California, USA, in 1969. Since then, EV71 has been identified globally. The clinical spectrum of EV71 infection ranges from asymptomatic infection, to mild hand-foot-mouth disease (HFMD), and severe cases with central nervous system (CNS), and cardiopulmonary involvement [1], [2]. Recent studies have further demonstrated that CNS-complicated EV71 infections could cause long-term cognitive and motor deficits [3], [4]. Globally, two patterns of EV71 outbreaks have been reported: small-scale outbreaks with few CNS-complicated cases and deaths, and large-scale outbreaks with frequent CNS-complicated cases and deaths [1]. The latter pattern occurred in Bulgaria, with 44 deaths in 1975 [5]; in Hungary, with 45 deaths in 1978 [6]; in Malaysia, with 29 deaths in 1997 [7]; in Taiwan, with 78 deaths in 1998 [8]; in Singapore, with 5 deaths in 2000 [9]; and recently in China, with more than 100 deaths in 2007, 2008, and 2009 [10]–[12]. Since the 1998 epidemic, EV71 has continued to cause nationwide epidemics again in 2000–2001, 2004–2005, and 2008–2009 in Taiwan [12]–[22].

No antiviral against EV71 is currently available, so development of EV71 vaccines has become a national priority in Taiwan and China, and several organizations in Asia are planning clinical trials of EV71 vaccines [12]. To design clinical trials of EV71 vaccines, age-specific incidence rates of EV71 infections are required to identify target populations, estimate disease burdens, select endpoints of clinical efficacy, and estimate sample size. Taiwan has had a national surveillance system for severe enterovirus infections since 1998. Age-specific incidence rates of EV71-related severe infections during the 1998 epidemic have been estimated to be 27.3, 37.1, 30.0, and 23.1 per 100,000 for children aged <6, 6–11, 12–23, and 24–35 months, respectively [23], which are too low to be a suitable clinical endpoint. Alternatively, EV71-related mild infections such as herpangina and HFMD could be suitable clinical endpoints; but their age-specific incidence rates are not available in Taiwan. We initiated a longitudinal cohort study in 2006 to estimate age-specific incidence rates of EV71 infection in young children in northern Taiwan.

## Methods

### Ethics statement

Institutional review board approval was obtained from **Chang Gung Memorial Hospital** (CGMH) following the Helsinki Declaration; and written informed consent was obtained from all mothers of participating infants.

### Study populations

Pregnant women having prenatal exams in CGMH were invited to participate in the study starting in June 2006. Sera were obtained from participating pregnant women and their children for measuring EV71 neutralizing antibody titers in the following schedule: pregnant women immediately before delivery; neonates at birth (cord blood); and infants at 6, 12, 24, and 36 months of age. About 20–30 participants were recruited every month. Parents were educated to contact study staff when their children developed suspected enterovirus illnesses (herpangina or HFMD). During enterovirus seasons, study staff actively contacted parents about suspected enterovirus illnesses by e-mail, telephone or text message. If the participating children developed suspected enterovirus illnesses, throat swabs were collected from these participating children for virus isolation. In previous studies, throat swabs were more sensitive than rectal swabs or feces for isolating EV71 viruses in acute EV71 cases [12]. Occasionally, pediatricians also collected throat swabs from the participating children who developed non-specific febrile illness for virus isolation during enterovirus seasons. We chose CGMH as a study site because it has large obstetric and pediatric populations and serves residents from both rural and urban areas in northern Taiwan [14], [22]–[24]. In additional to the infant cohort study, clinical samples (throat swabs, rectal swabs, or stool samples) are routinely collected for virus isolation from hospitalized pediatric patients with suspected enterovirus infections (herpangina, HFMD, or non-specific febrile illness) in CGMH. Monthly distribution of EV71 isolation in pediatric inpatients the study hospital is based on the routine statistics and is anonymized. This information was used to decide EV71 seasons for data analysis. Data related to serum EV71 antibody titers in pregnant women, neonates and 6-month-old infants have been published previously [24] and this report focused on incidence rate of EV71 infections in the first three years of life.

### Clinical and laboratory definitions

In this study, evidence of herpangina included oral ulcerations on anterior tonsillar pillars, soft palate, buccal mucosa, or uvula. Evidence of HFMD included oral ulcers on the tongue and buccal mucosa, and a vesicular rash on the hands, feet, knees, or buttocks. Nonspecific febrile illness was defined as a rectal temperature greater than 38°C without other symptoms. Laboratory evidence of EV71 infection was defined as the isolation of EV71 from a throat swab; or a ≥4-fold rise or seroconversion in EV71 neutralizing antibody titers in paired sera samples. Asymptomatic EV71 infection was defined as a seroconversion in EV71 neutralizing antibody titers in paired sera samples without display of clinical symptoms during the period of collection of paired sera.

### Virological analysis

Samples were inoculated into human embryonic fibroblast, LLC-MK2, HEp-2, and rhabdomyosarcoma cell cultures. When enteroviral cytopathic effect involved more than 50% of the cell monolayer, cells were scraped and subjected to indirect fluorescent antibody staining with enterovirus monoclonal antibodies [25].

### Serologic assays

Laboratory methods for measuring EV71 serum neutralizing antibody titers followed standard protocols [26]–[27]. Twofold serially diluted sera (1∶8–1∶512) and a virus working solution containing 100 TCID_50_ of EV71 strain E59-TW-2002 (B4 genotype) were mixed on 96-well microplates and incubated with rhabdomyosarcoma cells. A cytopathic effect was observed in a monitor linked with an inverted microscope after an incubation period of 4 to 5 days. The neutralization titers were read as the highest dilution that could result in a 50% reduction in the cytopathic effect. Each test sample was run simultaneously with cell control, serum control, and virus back titration. The starting dilution was 1∶8; the cutoff level of seropositivity was set at 8. For deciding serostatus (positive or negative), sera were tested only at 1∶8.

### Statistical analysis

Since no EV71 infection was detected in our study cohort in 2006 and 2007 [22], observation time (person-months) for participants who returned for follow up during 2008 and 2009 were summed and converted to person-years to calculate age-specific incidence rates and 95% confidence interval (95% CI) based on the Poisson distribution [27]. Cumulative incidence rates were calculated for participants who returned for follow up; and 95% CIs of cumulative incidence rates were calculated based on the binominal exact distribution. Neutralization antibody titers were log-transformed to calculate the GMT and 95% CI. The statistical association between two nominal or ordinal variables was tested by the χ^2^ test, Fisher's exact test, or the Mantel-Haenszel χ^2^ test for trend, as appropriate. All statistical analyses were performed using Microsoft Excel (Microsoft, Redmond, WA, USA) or SAS (SAS Institutes, Cary, NC, USA).

## Results

### Enterovirus Surveillance

**Figure 1:**
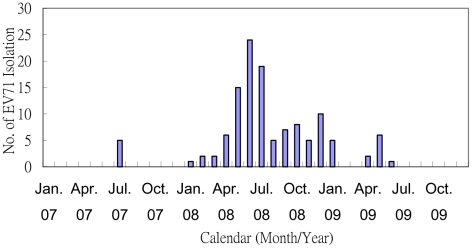
Enterovirus 71 isolation in pediatric inpatients in Chang-Gung Memorial Hospital, Taiwan, 2007–2009.

In CGMH, clinical samples are routinely collected for virus isolation from hospitalized pediatric patients with suspected enterovirus infections. From 2007 to 2009, 4691 pediatric inpatients were tested for enterovirus cultures and the enterovirus isolation rates in these three years were 14% (207/1475), 21% (420/1969), and 15% (181/1247), respectively. EV71 was isolated from 123 inpatients, including 5 cases in 2007, 104 cases in 2008, and 14 cases in 2009. Only 5 sporadic EV71 inpatients were detected in 2007, all in the middle of the year. In January 2008, EV71 inpatients began being detected again, with the numbers growing rapidly before peaking in June of that year and disappearing after July 2009 (Fig. 1). Overall, the pattern was consistent with that observed in the prospective cohort study.

### Laboratory Diagnosis

Between June 2006 to June 2009, 749 neonates were recruited into the cohort study. In 2008–09, 124 herpangina and 9 HFMD cases were detected and 128 of them provided clinical samples (throat swabs or serum) for laboratory diagnosis. In addition, 20 children developing non-specific illnesses including fever, upper respiratory illness, and viral exanthema also provided clinical samples for laboratory diagnosis. Among 118 symptomatic children providing throat swabs for virus culture, 40 (34%) had enterovirus isolated and 5 were EV71. In total, 20 symptomatic EV71 infections were detected in the children's cohort study, including 5 herpangina cases, 6 HFMD cases, and 9 cases with the non-specific illnesses (Table 1). None of the EV71 cases identified in the cohort developed complications. Among the 20 symptomatic EV71 infections, all developed seroconversion and 9 of them provided throat swabs for virus culture in acute phase. In these 9 cases, EV71 and cytomegalovirus were isolated from 5 and 2 cases, respectively. Overall, the virus isolation rate was significantly lower than the seroconversion rates for detecting EV71 infections (4.2% vs. 16.7%, P<0.01, Fisher's exact test). In addition, 8 asymptomatic EV71 infections were detected by seroconversion.

**Table 1:** Laboratory diagnosis of symptomatic EV71 infections in young children, northern Taiwan, 2008–2009.

Clinical symptoms	No. of cases	Diagnosis of EV71 infections by different assays		
		Virus isolationn/N (%)	Serologyn/N (%)	Combinedn/N (%)
Herpangina	124	2/100 (2.0)	5/93 (5.4)	5/119 (4.2)
HFMD	9	3/6 (50)	6/9 (67)	6/9 (67)
Other*	20	0/12 ( 0)	9/18 (50)	9/20 (50)
Total	153	5/118 (4.2)	20/120 (17)	20/148 (16)

HFMD: hand-foot-mouth disease.

*Including febrile illness, upper respiratory illness, and viral exanthema.

### Incidence Rate

Between January 2008 and December 2009, 307, 391, 294, and 66 children returned for follow up and serum collections at 6, 12, 24, and 36 months of age, respectively. Overall, the follow-up rates at 6, 12, 24 and 36 months of age were 73%, 67%, 55% and 51%, respectively. The major reason for loss of follow-up was refusal of providing blood samples. As shown in Table 2, age-specific incidence rates of EV71 infections in young children increased gradually from 1.71 per 100 person-years at 0–6 months of age to 4.09, 5.74, and 4.97 per 100 person-years at 7–12, 13–24, and 25–36 months of age, respectively. Table 3 shows the cumulative incidence rates, which increased from 0.65% (95% CI, 0.08%, 2.33%) at 6 months of age to 6.46% (95% CI, 3.94%, 9.91%) and 15.15 (95% CI, 7.51%, 26.1%) at 24 and 36 months of age, respectively.

**Table 2:** Age-specific incidence rates of enterovirus 71 infection in northern Taiwan, 2008–09.

Age(months)	No. of persons (person-month) followed	No. of infections			Incidence rate#(95% CI$)
		Symptomatic	Asymptomatic*	Total	
0–6	307 (1407)	0	2	2	1.71 (0.21, 6.16)
7–12	391 (2052)	5	2	7	4.09 (1.65, 8.43)
13–24	294 (3343)	13	3	16	5.74 (3.28, 9.33)
25–36	66 (724)	2	1	3	4.97 (1.03, 14.53)
Total		20	8	28	

*Asymptomatic infection is identified based on seroconversion of EV71 neutralizing antibody in scheduled blood samples.

# per 100 person-years.

$ 95% confidence interval calculated based on the Poisson distribution.

**Table 3:** Cumulative incidence rates of enterovirus 71 infection in northern Taiwan, 2008–09.

Age of blood collection (months)	No. of children followed	No. of infections by age (months)					Cumulative incidence rate*(95% CI#)
		0–6	7–12	13–24	25–36	Total	
6	307	2				2	0.65 (0.08, 2.33)
12	391	1	7			8	2.05 (0.89, 3.99)
24	294		3	16		19	6.46 (3.94, 9.91)
36	66			7	3	10	15.15 (7.51, 26.10)

*per 100 persons.

# 95% confidence interval was calculated based on the binominal exact distribution.

Two children who acquired EV71 infections before 6 months of age were both asymptomatic and one of them had neutralizing antibody titer in cord blood (antibody titer 16). Among 7 children who acquired EV71 infections at 7–12 months of age, 2 were asymptomatic and 5 were symptomatic. The 2 asymptomatic cases both had neutralizing antibody titer in cord bloods and their maternal antibodies declined to undetectable level at 6 months of age. In contrast, only 2 of the 5 symptomatic cases had detectable EV71 neutralizing antibody in cord blood and their maternal antibodies also declined to undetectable level at 6 months of age. The 2 symptomatic cases with detectable maternal antibody in cord blood were infected at 7 and 9 months of age and the 3 symptomatic cases without detectable maternal antibody in cord blood were infected at 7, 8 and 9 months of age.

## Discussion

EV71 continues to cause disease with the potential for life-threatening infections in Asian children. Development of EV71 vaccines has become a national priority in Taiwan and China. To design clinical trials of EV71 vaccines, age-specific incidence rates of EV71 infections are required to identify target populations and select study endpoints. In this study, risk of EV71 infections greatly increased after 6 months of age during EV71 epidemics, which is consistent with national surveillance data of severe EV71 infections and decline of maternal antibody by 6 months of age [23]–[24]. Furthermore, the cumulative incidence rate in young children was about 15% by 36 months of age after follow-up for 2 years, which is consistent with the results of seroprevalence studies conducted after the 1998 epidemic [23], [28]. The follow-up rate by 36 months of age was about 51% in our study but the major reason for loss of follow-up was refusal of providing blood samples. Meanwhile, 17 (61%) of the 28 EV71-infected children continue to return for follow-up. Overall, follow-up rates in EV71-infected and uninfected children differed slightly. Therefore, the substantial loss of follow-up in our study may not cause significant bias in calculation of incidence. However, our cohort study was conducted in northern Taiwan and more severe EV71 cases were reported in southern Taiwan than in northern Taiwan during the 2008–2009 epidemic. It should be cautious about extrapolating finding of this study to whole Taiwan. In addition, EV71 epidemics occurred every 3–4 years in the past 10 years in Taiwan, which may greatly affect the estimation of age-specific incidence rates.

In addition, in our prospective cohort 29% of EV71 infections in young children were asymptomatic, 32% had non-specific illness, and 39% developed herpangina/HFMD. In a retrospective study conducted in Taiwan in 1999, Chang et al. found that 29% of 484 EV71-seropositive children <6 years of age developed herpangina/HFMD and 71% were asymptomatic [23]. In a prospective hospital-based case-finding study conducted in a medical center in 2001–02, 6% of 183 EV71 infections in children <18 years of age were asymptomatic, 13% developed non-specific illnesses, and 71% developed herpangina/HFMD [14]. It is hard to compare our study with the other two studies due to differences in study designs, age groups, laboratory diagnosis, and genotypes of circulating EV71. Overall, the retrospective study could not differentiate EV71 infections with non-specific illnesses from asymptomatic EV71 infections and the prospective hospital-based case-finding study would underestimate the proportion of asymptomatic infections. Therefore, our prospective infant cohort study is more likely to reflect occurrences of EV71 infections in the community.

Due to small sample size, our prospective study could not detect any complicated EV71 infections. In the prospective hospital-based case-finding study, 21% of 183 EV71 infections in children <18 years of age developed neurological complications such as meningitis and encephalitis [14]. Based on national severe enterovirus surveillance with virus isolation and two cross-sectional serosurveys, Lu et al. estimated that 130617 Taiwanese children aged <3 years were infected with EV71 infections in 1998 and that 273 (0.21%) of these infected children developed neurological complications [28]. Overall, the prospective hospital-based case-finding study would overestimate the proportion of EV71 infections with neurological complications and the national surveillance data would underestimate the proportion of EV71 infections with neurological complications.

Interestingly, we found that the 2 children who acquired EV71 infections before 6 months of age were both asymptomatic and their EV71 neutralizing antibody titers at birth, 6 and 12 months of age were 16, 256 and 32, and <8, 128 and no serum collected at 12 months of age, respectively. Chang et al. [23] also found that children <6 months of age were more likely to develop asymptomatic EV71 infections than children >6 months of age. These observations may indicate that maternal antibody may provide protection against symptomatic EV71 infections in young infants even though EV71-specific maternal antibody may have declined to undetectable levels in these young infants.

Current standard methods for laboratory diagnosis of EV71 infections include virus isolation and serum neutralizing antibody testing. In the hospital-based case finding study, serum neutralizing antibody testing (≥4-fold rise) was more sensitive than virus isolation for detecting EV71 infections [12], which was consistent with the finding of our infant cohort study. However, the serum neutralizing antibody testing needs to collect paired sera, which are not always available. Several studies have found that serum IgM tests based on single clinical specimen were more sensitive than virus isolation for detecting EV71 infections but had a major drawback of high false positives, especially for patients infected with Coxsackievirus A16 [29], [30]. In addition, molecular tests based on single clinical specimen were also more sensitive than virus isolation for detecting EV71 infections but had a major limitation of high cost [31]–[33]. Our cohort study collected serial sera to detect EV71 infections and would also be more sensitive than molecular tests based on a single clinical specimen. Overall, enterovirus surveillance systems based only on virus isolation for detecting EV71 infections would significantly underestimate disease burdens of EV71 infections [7]–[9]. However, after introducing vaccines, vaccinated children would possess EV71 antibodies which would confound serological diagnosis of EV71 infection so it would be necessary to detect and serotype enteroviruses using virus isolation and molecular methods. Overall, harmonized and integrated laboratory diagnosis methods are urgently needed to make national enterovirus surveillance data comparable [12].

Like poliomyelitis viruses, vaccination would be the most cost-effective intervention to prevent EV71-related diseases in endemic countries. In Taiwan, the target population of EV71 vaccines has been identified to be infants <6 months of age [23], [24]. Our prospective infant cohort study has found that EV71-related HFMD/herpangina is a suitable endpoint of vaccine efficacy trials in Taiwan. In addition, the cumulative incidence rate of EV71 infections during an EV71 epidemic was about 15% by 36 months of age, and 29% of EV71 infections were asymptomatic in young children, that are critical for designing vaccine clinical trials. Several organizations are planning clinical trials of EV71 vaccines in Asia; and EV71 vaccines could be available in the near future [12]. To successfully introduce EV71 vaccines in epidemic areas, each country needs to well characterize its EV71 epidemiology [34], [35]. Our prospective infant cohort study would be helpful to other countries for understanding their EV71 epidemiology.

## Supporting Information

Checklist S1 **STROBE checklist.**
(DOC)
